# A New Frequency Analysis Operator for Population Improvement in Genetic Algorithms to Solve the Job Shop Scheduling Problem †

**DOI:** 10.3390/s22124561

**Published:** 2022-06-17

**Authors:** Monique Simplicio Viana, Rodrigo Colnago Contreras, Orides Morandin Junior

**Affiliations:** 1 Department of Computing, Federal University of Sao Carlos, Sao Carlos 13565-905, SP, Brazil; orides@ufscar.br; 2 Department of Computer Science and Statistics, Institute of Biosciences, Letters and Exact Sciences, Sao Paulo State University, Sao Jose do Rio Preto 15054-000, SP, Brazil; contreras@usp.br; 3 Department of Applied Mathematics and Statistics, Institute of Mathematical and Computer Science, University of Sao Paulo, Sao Carlos 13566-590, SP, Brazil

**Keywords:** evolutionary algorithm, genetic algorithm, genetic improvement, job shop scheduling problem, combinatorial optimization

## Abstract

Job Shop Scheduling is currently one of the most addressed planning and scheduling optimization problems in the field. Due to its complexity, as it belongs to the NP-Hard class of problems, meta-heuristics are one of the most commonly used approaches in its resolution, with Genetic Algorithms being one of the most effective methods in this category. However, it is well known that this meta-heuristic is affected by phenomena that worsen the quality of its population, such as premature convergence and population concentration in regions of local optima. To circumvent these difficulties, we propose, in this work, the use of a guidance operator responsible for modifying ill-adapted individuals using genetic material from well-adapted individuals. We also propose, in this paper, a new method of determining the genetic quality of individuals using genetic frequency analysis. Our method is evaluated over a wide range of modern GAs and considers two case studies defined by well-established JSSP benchmarks in the literature. The results show that the use of the proposed operator assists in managing individuals with poor fitness values, which improves the population quality of the algorithms and, consequently, leads to obtaining better results in the solution of JSSP instances. Finally, the use of the proposed operator in the most elaborate GA-like method in the literature was able to reduce its mean relative error from 1.395% to 0.755%, representing an improvement of 45.88%.

## 1. Introduction

Combinatorial optimization problems (COPs) consist of situations in which it is necessary to determine, through permutations of elements of a finite set, the configuration of parameters that is more advantageous [1]. Due to its high degree of applicability, many researchers have been using COPs in different contexts—for example, applications in the logistics [2], vehicle routing [3] and railway transport control [4], among other current problems [5]. In particular, one of the most addressed COPs in the literature is production scheduling [6], which, according to Groover [7], is part of the Production Planning and Control activities and is responsible for determining the design of operations that will be conducted, such as the environment in which products are processed, what resources are used and what is the start and end time for each production order.

Academic research and the development of solution methodologies have focused on a limited number of classic planning and production scheduling problems, one of the most researched is the variation known as Job Shop Scheduling Problem (JSSP) [8], in which a finite set of jobs must be processed by a finite set of machines. In this category of problems, the objective is usually to determine a configuration in the order of processing of a set of jobs or tasks to minimize, for example, the time of using resources [9]. In this case, several performance measures are useful to evaluate how satisfactory a given configuration is for a JSSP, with a makespan [10] that corresponds to the total time needed to finish the production of a set of jobs (one of the most used).

Belonging to the well-known class of problems NP-Hard, JSSP presents itself as a computational challenge, since it is not a trivial task to develop an approach to determine exact solutions that represent a configuration with an adequate performance measure, within a reasonable time, even considering small and moderate cases [11]. From this need, algorithms that present approximate results in a feasible computational time were developed and applied to JSSP. The main methods used are those composed of meta-heuristics [12], mainly by the Evolutionary Algorithm (EA) known as Genetic Algorithm (GA) [13,14,15,16,17]. Even so, the JSSP consists of a class of problems that remain open [18] and with many instances still unsolved in the well-known benchmarks of the area [19]. This is because the existing methods do not have the necessary efficiency to guarantee their practical use.

More specifically, it is possible to highlight certain disadvantages in the use of GA in solving COPs [20,21]. In detail, it is common for this set of techniques to become stagnant [22], during their iterations in solutions that are local minimums, which configures the phenomenon known as premature convergence [23]. Furthermore, GAs may require high computational time [24] to obtain good solutions to this type of problem. Therefore, for complex problems, GA needs to be assimilated to specific problem routines to make the approach effective. Hybridization can be a deeply effective way to improve the performance of these techniques. The most common form of hybridization is the addition of GAs to local search strategies and the incorporation of domain-specific knowledge in the search process [25].

In the latter, there are genetic improvement operators through manipulations in specific genes on a chromosome. These have a main objective to provide reinforcement coming from one or more individuals who have been successful in the adaptation process to individuals who are not able to stand out in a population. In other words, these operators direct the worst individuals in a population to areas known to be good in the search space.

The authors do Amaral and Hruschka JR [26,27] presented an operator in this line of reasoning, entitled a transgenic operator, which simulates the process of genetic improvement. To conduct such a procedure, in one of the stages of the GA, the population of the same is replicated to four parallel sub-populations, and in each of these four populations, the best individuals transfer up to four genes, based on historical information, to selected individuals. Then, only the best individuals among the four sub-populations remain.Viana, Morandin Junior and Contreras [15] proposed an adaptation of the transgenic operator of do Amaral and Hruschka JR [27] to solve a JSSP with GA. The authors propose the identification of relevance in the genes used in the transgenic process through a preprocessing step. However, such preprocessing is computationally time-consuming and may not be viable in large JSSPs.

In this work, we propose a new population guidance operator for GAs: the Genetic Improvement based on Frequency Analysis (GIFA) Operator. Our method consists of a new way to determine the genetic relevance based on the frequency analysis of the genes of individuals who have good fitness values in the population. We also propose the construction of a representative individual that represents this group of good individuals and that is used in the process of genetic manipulation to guide the worst individuals towards good solutions and, potentially, that these become positive highlights in the population.

This paper is an extended version of our preliminary work [28]. In this manuscript, we add a literature review section, and we consider more testing instances in our experimental evaluations. Furthermore, all steps of the method are outlined and detailed in the form of algorithms that simplify the reproducibility of the technique. This work is divided into six sections. Specifically, we discuss, in Section 2, works related to ours. In Section 3, we describe the JSSP fundamentation. We present, in Section 4, the details about the proposed GIFA operator and the requirements that a GA needs to satisfy to use it. Experimental results on different GAs using GIFA and the advancement in the state of the art of JSSPs are presented in Section 5. The work is finished in Section 6 with conclusions about the developments as well as future projections for improving the method and possible applications.

## 2. Related Works

Several meta-heuristics have been proposed in the literature to treat the JSSP, such as GA [11,13,15,20,29,30,31,32,33,34,35]; Simulated Annealing [36,37]; Hybrid social spider optimization [38]; Harris hawk optimizer [39]; Grey Wolf Optimization [40]; Bat Algorithm [41]; Chicken Swarm Optimization [42], Single seekers society:[43] and Particle Swarm Optimization: [44]. However, GAs remain one of the most common approaches used in resolving JSSPs. In the following paragraphs, we discuss certain works in the literature that deal with the JSSP production scheduling problem through meta-heuristics. These works were chosen because they have a great impact on the specialized literature and/or represent the state-of-the-art. The following detailed works are those authored by Ombuki and Ventresca [29], Watanabe et al. [30], Asadzadeh [20], Jorapur et al. [31], Wang et al. [11], Wang et al. [36], Dao et al. [41], Jiang [40], Semlali et al. [42] and Kurdi [32].

The authors Ombuki and Ventresca [29] proposed the Local Search Genetic Algorithm (LSGA) meta-heuristic to treat JSSP. The proposed LSGA is a Genetic Algorithm (GA) with local search, which has an operator similar to the mutation that is focused on local research, with the aim of further improving the quality of the solution. The LSGA of Ombuki and Ventresca [29] is a hybrid strategy that uses GA with the addition of a Tabu Search (TS) routine. LSGA was one of the first works to incorporate a more elaborate local search strategy, which proved to be efficient in the GA-like methods of the time. However, the technique was not able to find the optimal values of medium difficulty instances, such as FT10 [45].

Watanabe et al. [30] proposed a meta-heuristic based on a modified GA with search area adaptation (GSA). The proposed GSA has an adaptation of the search area with the ability to adapt to the structure of the solution space and to control the balance between global and local searches. The crossover operation of the GSA consists of performing the crossover several times on all pairs of parents each time a new cutoff point is drawn. The crossover is repeated until a child better than the worst individual in the population is found or until a certain number of iterations is reached. The GSA mutation operation consists of executing perturbations several times on all children and performing several swaps in their genes. The mutation is repeated until a mutant child better than the worst individual in the population is found or until a certain number of iterations is reached. As it was one of the first methods in this sense, the GSA was evaluated in a few instances and presented results far below the most recent methods.

On the same theme, Asadzadeh [20] presented the meta-heuristic Local Search Genetic Algorithm (aLSGA) with the inclusion of intelligent agents. The method is composed of a multi-agent system, in which each agent has a specialized behavior to implement the local search. The aLSGA combines local search heuristics with crossover and mutation operators. The use of multiple mutation functions expands the search power of the method; however, for the more elaborate search strategy of the method, only one function is considered in aLSGA.

The authors Jorapur et al. [31] proposed the Promising Initial Population-Based Genetic Algorithm (IPBGA) meta-heuristic. The IPBGA algorithm is a combination of GA with a new job-based modeling for the construction of the initial population. The objective of the work was to present an alternative population modeling for GA and, also, to present the impact that this type of alteration can obtain concerning the effectiveness of GA. However, in addition to the IPBGA achieving the best-known solution in few instances; in the others, the results obtained were significantly far from the optimal solution.

The Adaptive Multi-population Genetic Algorithm (AMGA) meta-heuristic was proposed by Wang et al. [11]. The  idea of AMGA is based on an GA that uses multi-population and has an adaptive probability of crossover and mutation, intending to expand the scope of the search and improve its performance. The work has some points that differ from other works that deal with the JSSP problem with GA. The first point is the insertion of multi-population in GA, the second point is to have an adaptive probability of crossover and mutation and the third point is that the elite individuals (individuals with better fitness) from each population are directly evolved into the next generation.

AMGA was tested on 39 instances, and it was able to find the best-known solution in 38 of those instances. The computational results showed that the AMGA can produce optimal or near-optimal values in almost all the benchmark instances tested; however, in the instances of Lawrence [46], not all were tested, and the instances that were left without evaluation are precisely the instances that present a greater complexity.

Jiang [40] developed the Hybrid Gray Wolf Optimization (HGWO) meta-heuristic. The HGWO is composed of the combination of the GWO algorithm with the local VNS algorithm as well as the addition of genetic operators (crossover and mutation) to balance the capacity of local and global exploration of the algorithm. In the proposal, three neighborhood structures were used: Swap, Insert and Inverse. The proposed algorithm obtained competitive results when compared with relevant works in the literature; however, of the 40 instances proposed by [46], only the 20 smallest were considered, and thus there is no way to evaluate the behavior of the algorithm in instances with greater complexity.

Wang et al. [36] proposed the TSAUN meta-heuristic, which is a hybrid local search algorithm. TSAUN is composed of the combination of the Simulated Annealing (SA) method and the Tabu Search (TS) method. The TSAUN structure runs an SA core and applies the TS technique to a local search. This hybrid algorithm takes advantage of stochastic SA to escape local minimums, and at the same time, improves the search performance through a TS. TSAUN did not achieve the best results in the tested instances; however, the method proved to be competitive with other works present in the state of the art. The work presents a contribution in the area of hybrid algorithms with the insertion of local search techniques, and  through the results obtained, the improvement that these combinations of techniques can achieve is reinforced.

In the article of Dao et al. [41], the meta-heuristic Parallel Bat Algorithm (PBA) was proposed, which is composed of the meta-heuristic Bat Algorithm (BA) with the inclusion of parallel processing. The objective of adding parallel processing to BA was that, with communication strategies, it is possible to correlate individuals in each cluster and share information among them. Communications provide improved diversity and accelerate the search for satisfactory solutions. Neighborhood operators of the types Swap, Insert and Inverse were also included in the proposal. It is clear from the work that the BA with the inclusion of parallel processing can achieve better solutions in JSSPs than can a basic BA.

Semlali et al. [42] proposed the meta-heuristic Memetic Chicken Swarm Optimization (MeCSO). The method integrates the Chicken Swarm Optimization (CSO) algorithm with local search method 2-opt of Croes [47]. The CSO algorithm was established by Meng et al. [48] and was inspired by the behavior of a swarm of chickens while looking for food. The algorithm had good efficiency in instances of smaller sizes; however, in larger instances, the method presented a great deal of difficulty. In this case, in observing the results, it is possible to notice that the algorithm has a tendency to become stuck in local optima and cannot go beyond certain points when considering larger instances.

Kurdi [32] investigated the impacts of selecting the genetic materials exchanged during the crossover with prior information about the critical paths that exist in the domain rather than randomly selecting them. Through the presented results, the author was able to present the impact that this area of study brings. According to the author, the basic proposed idea for the identification of the genes that hold the most important characteristics is a promising area of research and deserves further investigation since it produces significant improvements when applied in the JSSP.

Hamzadayı et al. [43] proposed to adapt some components of the Single Seekers Society (SSS) metaheuristic to deal with combinatorial optimization problems. The proposed SSS was applied in two types of production scheduling: the Flow Shop Scheduling Problem and the JSSP. The SSS algorithm consists of a metaheuristic that allows cooperation between different search heuristics. In this case, SSS incorporates several metaheuristics, such as Simulated Annealing, Threshold Accepting, Greedy Search (GS), and all information from each method works in an integrated way. To generate new solutions, SSS shares information via crossover and handles the search by integrating the information via neighborhood structure. SSS was not the method that obtained the best results for JSSP instances; however, it obtained competitive results and was able to find the best known value in 14 instances of the 20 instances that were tested. The method proved to be able to maintain its effectiveness in similar combinatorial optimization problems, obtaining satisfactory results in both the production and scheduling problems evaluated.

Yu et al. [44] proposed an improved hybrid PSO with non-linear inertia weight and Gaussian mutation (NGPSO) to solve the JSSP. The nonlinear inertia weight was added to the method in order to improve the local search capability and the Gaussian mutation was added in order to improve the global search capability. The method seeks to maintain a balance between local searches and ensuring population diversity, thereby, reducing the probability of the algorithm falling into a local optimal solution. The experimental results indicated that the NGPSO algorithm had satisfactory performance and high capacity in JSSP resolution, and was able to find the best known value in 38 of 62 instances. The techniques added to improve local search and global search significantly improved the PSO; however, for more complex instances, a better balance between searches is needed.

A hybrid discrete Cuckoo Search (CS) method with Simulated Annealing, called DCSA, was proposed by Alkhateeb et al. [49] to handle JSSP instances. DCSA incorporates the SA optimization operators into the CS search algorithm. A combination of VNS and Lévy flight methods is used for a better exploration of the search space. In the performed evaluations, the DCSA presented a faster convergence than the other compared methods and was able to find the best known solution in 29 instances of the 34 that were selected for testing. The DCSA also presented a lower computational cost compared with the other methods compared. The authors assumed that the improvement must be attributed to the integration of SA to CS and to the use of different exploration methods, such as VNS and Lévy flight. However, in the work, the instances considered more difficult were not considered, and  most of the methods in the literature usually became stuck in local minima.

In the works of Viana et al. [13] and Viana et al. [14], a new GA approach with improved local search and multi-crossover techniques (mXLSGA) was proposed. Three operators specialized in local search were proposed: one built into the mutation operator; one with massive behavior; and another with multi-crossover routines. Viana et al. [15] proposed a genetic algorithm with the inclusion of an operator called “Transgenic”. This operator is based on the idea of genetically modified organisms and with the proposal to guide individuals, who have the worst fitness values in the population, to a region of the search space that would be more favorable for solving the problem.

This operator selects significant genes from individuals that have been well evaluated and inserts those genes into the worst individuals through a preprocessing step in the form of JSSP resolution simulations. In this work, we propose an alternative to the transgenic operator in the sense that a preprocessing step, which is usually expensive, is unnecessary, since the calculation of gene importance is performed during each generation of the method.

We can see through the bibliographic review that JSSP has attracted the attention of several researchers due to having combinatorial behavior and being classified as NP-Hard. Several approaches using meta-heuristics applied in JSSP have been proposed, and some have included intelligent agents, parallel populations or the hybridization of meta-heuristics with other techniques. It appears, through the works reported, that hybridization is an effective way to improve the performance and effectiveness of several meta-heuristics. Some forms of hybridization successfully applied in the literature are the union of local search strategies and the incorporation of specific knowledge of the domain in the search process.

## 3. Formulation of the Job Shop Scheduling Problem

We can define JSSP as a COP that has a set of *N* jobs that must be processed on a set of *M* machines. Furthermore, each job has a script that determines the order in which it must pass through the machines for its process to be completed. Each job processing per machine represents an operation and the objective of a JSSP can be interpreted as being the challenge of determining the optimal sequencing of operations with one or more performance measures as a guide. The components of this problem follow certain restrictions [9]:

- Each job can be processed on a single machine at a time.
- Each machine can process only one job at a time.
- Operations are considered non-preemptive, i.e., they cannot be interrupted.
- Configuration times are included in the processing times and are independent of the sequencing decisions.

In this work, we adopted makespan (MKS) as a performance measure. The MKS is the total time that a JSSP instance takes to complete the processing of a set of jobs on a set of machines considering a given operation sequence.

Mathematically, let us assume the following components of a JSSP:

- $J=\{J_{1},J_{2},\cdots ,J_{N}\}$ is the set of jobs.
- $M=\{m_{1},m_{2},\cdots ,m_{M}\}$ is the set of machines.
- $O=(O_{1},O_{2},\cdots ,O_{N\cdot M})$ is an operation sequence that sets the priority order for processing the set of jobs in the set of machines.
- $T_{i}\left(O\right)$ represents the time taken by the job $J_{i}$ to be processed by all machines in its script according to the operation sequence defined in *O*.

Then, according to [13,14,15], the MKS can be defined as the total time that all jobs take to be processed according to a given operation sequence, as presented in Equation (1).

(1) $$\mathrm{MKS}=\max_{i}T_{i}\left(O\right).$$

It is worth mentioning that, in this work, a more intuitive notation was adopted for modeling the JSSP constraints and measures. However, mathematically elaborate formulations involving constrained optimization can be found in the specialized literature. For that, we suggest the survey of Xiong et al. [50] to the interested reader.

## 4. A New Genetic Improvement Operator Based on Frequency Analysis for GA Applied to JSSP

In this section, we present in detail how the proposed method works. We specify the idea of determining genetic relevance by analyzing the frequency of genes that represent good characteristics in individuals with adequate fitness values in the population and, with that, we intend to obtain innovation with the following three topics:

- A new strategy for defining genetic relevance in GAs chromosomes.
- A new genetic improvement operator that is versatile and can be used in GA variations.
- Improving the state of the art of JSSP benchmark results.

### 4.1. Genetic Representation

Our operator was developed to operate in all GA-like methods with minor modifications. In the meantime, we conduct its fundamentation on a specific encoding. In this case, we use the “coding by operation order” [51]. In this representation [13], the feasible space of a JSSP instance defined by *N* jobs and *M* machines is formed by chromosomes $c\in N^{N\cdot M}$, such that exactly *M* coordinates of *c* are equal to *i* (representing the job index *i*), for every i∈{1,2,⋯,N}.

This encoding determines in the chromosome the operation priority with respect to the machine allocation. For example, as in [14], let us assume c=(2,1,2,2,1,1) as a feasible solution in a JSSP instance with dimension 2×3 (N=2 and M=3). Thus, according to the operations defined in *c*, the following actions must be conducted in parallel or if the previous action has already been completed:

- (First) Job 2 must be processed by the first machine of its script.
- (Second) Job 1 must be processed by the first machine of its script.
- (Third) Job 2 must be processed by the second machine of its script.
- (Fourth) Job 2 must be processed by the third machine of its script.
- (Fifth) Job 1 must be processed by the second machine of its script.
- (Sixth) Job 1 must be processed by the third machine of its script.

### 4.2. Fitness Function

The encoding used makes it natural to define the fitness function of the problem as the makespan of a JSSP instance given according to the stipulated operation sequence—that is, the fitness function [15] used is given according to Equation (2):

(2) $$\begin{array}{cccc} F: & O & ⟶ & R\\ \; & O & ⟼ & F\left(O\right):=\max_{i}T_{i}\left(O\right), \end{array}$$

in which O is the set of all possible operation sequences for the defined JSSP instance.

In this way, for this fitness function, the MKS of the JSSP instance is calculated according to a given operation sequence, and then the meta-heuristic must look for an operation sequence in which the MKS is as small as possible and, consequently, the set of jobs must be processed by the set of machines taking the shortest possible time.

### 4.3. Proposed Genetic Improvement Based on Frequency Analysis Operator

In this work, we propose a new genetic improvement operator for evolutionary algorithms: the GIFA operator. The operator is based on a frequency analysis matrix calculated during the iterations of each GA. GIFA aims to calculate which genes on a chromosome can direct individuals with poor fitness values to better solutions and better search spaces. GIFA has two main stages: the first being defined by the making of the representative individual—that is, an individual that is determined by the configuration of the most frequent genes in the best individuals in the population; and the second stage consists of the use of the representative individual in the transgenic process—that is, the genetic manipulation through the insertion of specific genes of the representative individual in genes of the worst individuals in the population. Below, we present these steps in detail.

**Stage 1: Composition of the representative individual** Initially, a portion of the population that presents the best fitness values is selected. Specifically, we select $N_{\mathrm{Top}}$ individuals who are considered to be good examples of solutions in the population. This selection is made according to an order based on the fitness value of individuals in the population, as presented in Algorithm 1.


**Algorithm 1** Defining the $N_{\mathrm{Top}}$ best individuals.
**Input:**
$N_{\mathrm{Pop}}$          Number of chromosomes in the population$P=\left\{p_{1},p_{2},\cdots ,p_{N_{\mathrm{Pop}}}\right\}$   Population*F*            Fitness function$N_{\mathrm{Top}}$          Number of good individuals1: F:={}2: **for** i=1 to $N_{\mathrm{Pop}}$ **do**3:  $\omega_{i}:=F\left(p_{i}\right)$4:  $F:=F\cup \left\{\omega_{i}\right\}$5: **end for**6: $\left(\omega_{i_{1}},\omega_{i_{2}},\cdots ,\omega_{i_{N_{\mathrm{Pop}}}}\right):=f_{\mathrm{sort}}\left(F\right)$        ▹ The function $f_{\mathrm{sort}}\left(\cdot \right)$ ascending sorts the elements in a given set. In this case, $i_{1}$ is the index of the lowest fitness, $i_{2}$ is the index of the second-lowest fitness and so on, up to $i_{N_{\mathrm{Pop}}}$, which is the index of the highest fitness value.
7: $\left(p_{i_{1}},p_{i_{2}},\cdots ,p_{i_{N_{\mathrm{Pop}}}}\right)$ ▹ We arrange individuals according to their fitness, from the best to the worst.8: **for**j=1 to $N_{\mathrm{Top}}$ **do**9:  $c_{j}:=p_{i_{j}}$        ▹ Let us define the $N_{\mathrm{Top}}$ best individuals.10: **end for** **Output:**  $\left\{c_{1},c_{2},\cdots ,c_{N_{\mathrm{Top}}}\right\}$  $N_{\mathrm{Top}}$ best individuals


In the sequence, for each index job *i*, a frequency vector ${\vec{v}}_{i}\in R^{N\cdot M}$ is associated, in which the number of its occurrences is stored in each coordinate where the product *i* appears exactly at the position of this coordinate on the chromosomes selected for comparison. In Algorithm 2, the construction of vectors ${\vec{v}}_{i}$ is detailed.

**Figure 1:**
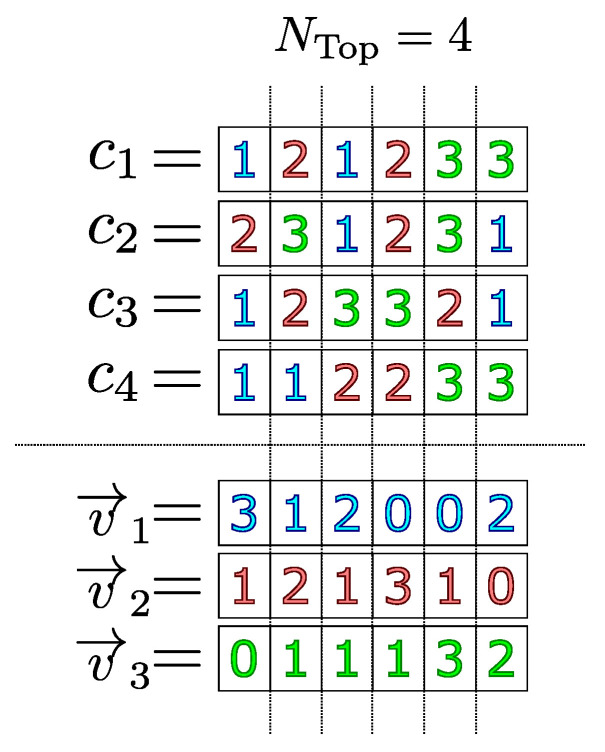
Calculating the frequency vectors (${\vec{v}}_{i}$) of the three jobs in each coordinate of the four best chromosomes in the population.

In Figure 1, an example of the calculation of the frequency vectors ${\vec{v}}_{i}$ is presented when considering four individuals $c_{1},c_{2},c_{3}$ and $c_{4}$ with the best values of fitness in a 3×2 dimension JSSP instance.


**Algorithm 2** Calculating the genetic frequency of the best adapted individuals.
**Input:**
$\left\{c_{1},c_{2},\cdots ,c_{N_{\mathrm{Top}}}\right\}$ $N_{\mathrm{Top}}$ best individualsN×M       Dimension of JSSP instance1: **for**
i=1 to *N* **do**2:  ${\vec{v}}_{i}:={\vec{0}}_{N\cdot M}$
▹ Initializing the frequency vectors. In this case, ${\vec{0}}_{N\cdot M}$ is the null vector with N·M coordinates.3: **end for**4: **for**
i=1 to *N* **do**5:  **for** j=1 to N·M**do**    ▹ The value N·M is the number of coordinates of chromosomes.6:   **for** k=1 to $N_{\mathrm{Top}}$ **do**7:    **if** $c_{k,j}=i$**then**       ▹ Is job *i* in the *j*-th coordinate of $c_{k}$?8:     $v_{i,j}:=v_{i,j}+1$
▹ If so, let us add 1 to the *j*-th coordinate of the frequency vector ${\vec{v}}_{i}$.9:     **end if**10:   **end for**11:  **end for**12: **end for** **Output:**  ${\vec{v}}_{1},{\vec{v}}_{2},\cdots ,{\vec{v}}_{N}$  Frequency vectors


Once the vector ${\vec{v}}_{i}$ has been made for every job index *i*, a chromosome whose coordinates are determined by the job with the highest frequency in this coordinate is defined as a representative individual. That is, each gene (coordinate) of the representative individual is defined as the job index that is most present in this coordinate in the best individuals in the population. It is also possible to establish an order of genetic relevance according to the frequency vectors ${\vec{v}}_{i}$.

That is, it is possible to define which genes of the representative individual are more suitable to be transferred in the process of genetic improvement. Such relevance is also defined according to the frequency that the products present in each coordinate of the best individuals so that the genes that present the same job in many good individuals can categorize a “trend” that leads to good fitness values. Therefore, these genes must be considered to be relevant, since they describe a positive characteristic in several individuals that stand out in the population. Mathematically, the representative individual and its genetic relevance are made according to the following procedure:

1. Let c be the representative individual and *w* a vector that designates a score for each of its coordinates, initially null. In the following items, the coordinates of c and *w* are made.
2. We define $I_{1}$ as being $\arg\max_{i}\left\{{\vec{v}}_{i,1}\right\}.$ That is, $I_{1}$ is the index of the job that has the highest frequency in the first coordinate of the exemplary individuals. Therefore, the first coordinate of the representative individual is defined as $I_{1}$. Mathematically,
  $$c_{1}:=I_{1}.$$
  In addition, a $w_{1}$ score defined as the maximum frequency shown in the first coordinate of the best individuals is associated with the first coordinate of c. That is,
  $$w_{1}:=\max_{i}\left\{{\vec{v}}_{i,1}\right\}={\vec{v}}_{I_{1},1}.$$
3. Assign the value 2 to *j*.
4. We define $I_{j}$ as being $\arg\max_{i}\left\{{\vec{v}}_{i,j}\right\},$ that is, $I_{j}$ is the most frequent product index in the *j* coordinate in the $N_{\mathrm{Top}}$ individuals. However, in order to guarantee the feasibility of the representative individual, it is necessary to establish two more restrictions:
  4.1 If the product $I_{j}$ is not in *M* coordinates of c, then it is defined as $I_{j}$ the *j*-th coordinate of the representative individual. That is,
    $$c_{j}:=I_{j}.$$
    In this case, the respective score is associated with the *j* -th coordinate of the representative individual as the maximum possible value presented in the *j*-th coordinate of the best individuals. That is,
    $$w_{j}:=\max_{i}\left\{{\vec{v}}_{i,j}\right\}={\vec{v}}_{I_{j},j}.$$
  4.2 Otherwise, to guarantee the feasibility of c, the frequencies of the index job $I_{j}$ are disregarded, since it is already arranged in *M* coordinates of c and, therefore, cannot occupy any more of its coordinates. To do so, we must cancel its respective frequency vector, that is,
    $${\vec{v}}_{I_{j}}:=\vec{0}.$$
    To make a new attempt, we must return to item 4.
5. If j≠N·M then j:=j+1 and we must return to item 4. Otherwise, the procedure is finished, and we have the representative individual pair and its respective genetic score (c,w).

**Figure 2:**
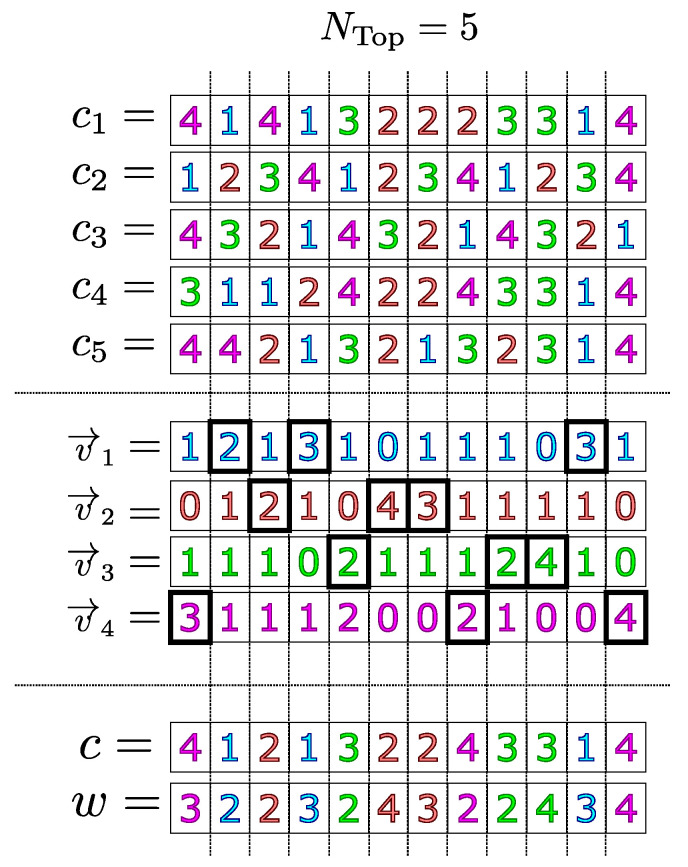
Computation of the representative individual (c) and its genetic relevance (*w*).

It is not necessary to project the representative individual in the feasible space of the problem since, due to its construction and the item 4 above, it is already feasible. In Figure 2, an example of the calculation of the representative individual (c) and the relevance of its genes (*w*) in a JSSP instance of dimension 4×3 is presented, taking, as the best individuals, the $N_{\mathrm{Top}}=5$ individuals with the lowest fitness values available in the population.

The details of the construction of the representative individual and the vector of relevance of its genes are presented in Algorithm 3.


**Algorithm 3** Calculating the representative individuals and their genetic relevance.
**Input:**
${\vec{v}}_{1},{\vec{v}}_{2},\cdots ,{\vec{v}}_{N}$   Frequency vectorsN×M       Dimension of JSSP instance1: $c={\vec{0}}_{N\cdot M}$        ▹ Initialize the representative individual as being null.2: $I_{1}:=\arg\max_{i}\left\{{\vec{v}}_{i,1}\right\}$ ▹ Most frequent job in the first coordinate of the best individuals.3: $w_{1}:=\max_{i}\left\{{\vec{v}}_{i,1}\right\}$  ▹ Number of times that the most frequent Job appears in the first coordinate of the best individuals.4: $c_{1}:=I_{1}$    ▹ Job $I_{1}$ occupies the first coordinate of the representative individual.5: j=2  ▹ Concerning the next coordinates, we proceed similarly but guaranteeing feasibility.6: **while**
j≤N·M
**do**7:  $I_{j}:=\arg\max_{i}\left\{{\vec{v}}_{i,j}\right\}$  ▹ Let us calculate the most recurring job in the *j*-th coordinate of the frequency vectors.8:  countJob:=0 ▹ To guarantee feasibility, each job must be in only *M* coordinates of the representative individual.9:  **for** k=1 to N·M **do**10:   **if** $c_{k}=I_{j}$ **then**11:    countJob:=countJob+112:   **end if**13:  **end for**14:  **if** countJob<M
**then**
▹ In case of feasibility, then we define the *j*-th coordinate of c.15:   $w_{j}:=\max_{i}\left\{{\vec{v}}_{i,j}\right\}$16:   $c_{j}:=I_{j}$17:   j=j+118:  **else**
▹ Otherwise, the next most recurring job in the *j*-th coordinate of the frequency vectors must be evaluated.19:   ${\vec{v}}_{I_{j}}={\vec{0}}_{N\cdot M}$
▹ Since $I_{j}$ makes c unfeasible, then we must disregard it for the next calculations.20:  **end if**21: **end while** **Output:**c  Representative individual*w*  Genetic relevance vector


#### 4.3.1. Stage 2: Use of the Representative Individual in Genetic Improvement

Once the representative individual and the relevance of each of its genes are calculated, then we propose that its most relevant genes be transferred to the worst individuals in the population, thus, simulating a mechanism for genetic improvement, or transgenics. For this, we take $P_{\mathrm{Worst}}:=\left\{x_{1},x_{2},\cdots ,x_{N_{\mathrm{Worst}}}\right\}$ as the set of the worst $N_{\mathrm{Worst}}$ individuals in a population. Subsequently, the most significant, or most relevant, $N_{\mathrm{Genes}}$ genes of the representative individual are transferred to all individuals in the $P_{\mathrm{Worst}}$ maintaining their original positions. This procedure can generate infeasible solutions.

Thus, it is necessary to conduct a correction, or projection, process on the individuals resulting from this operation. For this, we carry out the projection through the Hamming distance [52] modifying only the genes that were not received from the representative individual. In this way, the individuals generated in this procedure are projected on the feasible set of the problem, giving rise to the genetically improved individuals $P_{\mathrm{Improved}}=\left\{{\hat{x}}_{1},{\hat{x}}_{2},\cdots ,{\hat{x}}_{N_{\mathrm{Worst}}}\right\}$.

**Figure 3:**
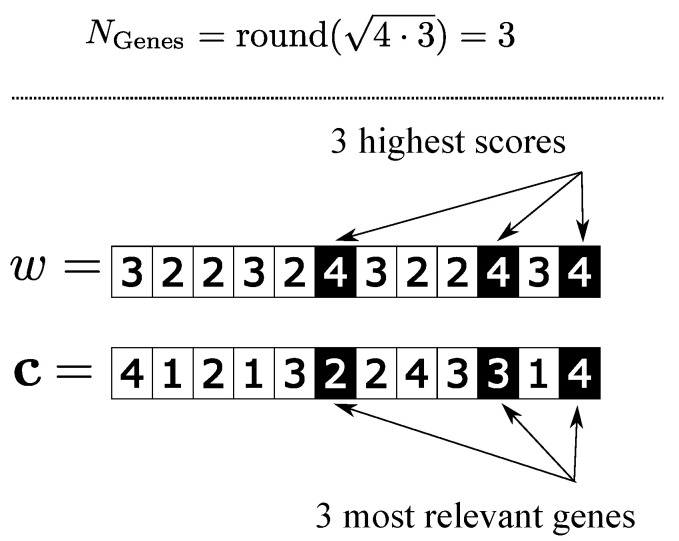
Determination of the most significant genes of a representative individual.

It is also necessary to establish how many genes will be transferred from the representative individual to the individuals of $P_{\mathrm{Worst}}$. For this, we follow a procedure similar to that of Viana, Morandin Junior and Contreras [15], which empirically determines that the adequate amount of genes used in the genetic improvement process is given by the root of the number of coordinates of the chromosome. Thus, the process remains advantageous and does not cause early convergence in the population. Thus, in this work, $N_{\mathrm{Genes}}$ is defined as $\mathrm{round}\left(\sqrt{N\cdot M}\right)$. In Figure 3, an example of the determination of the most significant genes of a representative individual c when it is given the scores of their genes *w* while addressing a JSSP with dimension 4×3.

In Algorithm 4, we present with comments all the steps of the proposed population improvement method.


**Algorithm 4** Population improvement using representative individual and genetic relevance.
**Input:**
c                  Representative individual*w*                 Genetic relevance vector$P_{\mathrm{Worst}}=\left\{x_{1},x_{2},\cdots ,x_{N_{\mathrm{Worst}}}\right\}$   The $N_{\mathrm{Worst}}$ worst individualsN×M              The JSSP dimension1: $N_{\mathrm{Genes}}:=\mathrm{round}\left(\sqrt{N\cdot M}\right)$        ▹ Number of genes to be transferred.2: **for**
i=1 to $N_{\mathrm{Genes}}$ **do**3:  $J_{i}:=arg\max_{k}\left\{w_{k}\right\}$    ▹c coordinates that represent its most important genes.4:  $w_{J_{i}}:=0$5: **end for**6: **for**
i=1 to $N_{\mathrm{Genes}}$ **do**7:  **for** k=1 to $N_{\mathrm{Worst}}$ **do**8:   $x_{k,i}:=c_{J_{i}}$     ▹Transferring the best genes from c to individuals in $P_{\mathrm{Worst}}$.9:  **end for**10: **end for**11: $P_{\mathrm{Improved}}:=\{\}$12: **for**
k=1 to $N_{\mathrm{Worst}}$ **do**13:  ${\hat{x}}_{k}:={\mathrm{proj}}_{\mathrm{Hamming}}\left(x_{k}\right)$ ▹ Correcting infeasible solutions with Hamming projection.14:  $P_{\mathrm{Improved}}:=P_{\mathrm{Improved}}\cup \left\{{\hat{x}}_{k}\right\}$15: **end for** **Output:**  $P_{\mathrm{Improved}}=\left\{{\hat{x}}_{1},{\hat{x}}_{2},\cdots ,{\hat{x}}_{N_{\mathrm{Worst}}}\right\}$  Improved individuals


**Figure 4:**
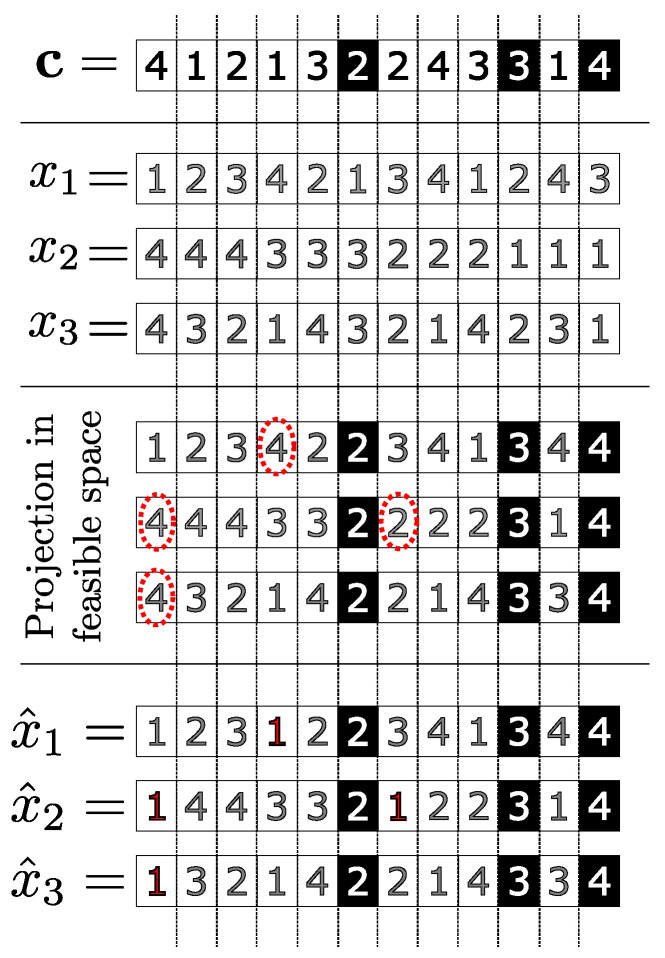
Genetic improvement proposed. The genes highlighted on a black background are the most relevant, while the genes highlighted with the red sectioned circle are those that need correction.

Assuming $N_{\mathrm{Worst}}=3$ and $P_{\mathrm{Worst}}=\left\{x_{1},x_{2},x_{3}\right\}$ as the set of the worst 3 individuals in a population, the improvement process is shown in Figure 4 genetic that transfers the $N_{\mathrm{Genes}}$ best genes from the representative individual c of to all individuals in the set $P_{\mathrm{Worst}}$.

The genetic improvement procedure must be performed after the standard operators of the GA, or the GA-like method used and right after the generation of a new population. Thus, the set $P_{\mathrm{Worst}}$ must be formed by individuals of the new population of the method. In addition, after applying genetic improvement, the evaluation of improvement or worsening of affected individuals is made so that the genetic changes made will only be saved in individuals who have obtained an improvement in fitness. That is, only individuals who have gained an advantage in the process of genetic improvement will be replaced in the population; the other individuals should be discarded and replaced by new individuals generated randomly as detailed in the next subsection.

#### 4.3.2. Generating New Individuals with the Lévy Flight Strategy

The proposed genetic improvement strategy was developed to be as versatile as possible in the sense that it can be attached to any GA-type method. Thus, the proposed operator (GIFA) must be used after the execution of the original operators of the considered algorithm in order to guide the solutions that were not able to stand out using such operators. In addition, the use of the proposed genetic improvement operator must be performed together with a genetic diversity maintenance strategy in order to not corroborate the premature genetic stagnation of the population.

One of the most commonly used routines in the literature for this purpose is the replacement of individuals from the population with new individuals generated from the Lévy distribution. Intuitively in [53], this distribution was associated with random walks whose steps are defined by exponential distributions—that is, $Lé\mathrm{vy}\left(s\right)∼{\left|s\right|}^{-1-\beta }$, with β∈(0,2]. Mathematically, as in [54], a random number generated by a Lévy distribution obeys the following distribution:

(3) $$Lé\mathrm{vy}\left(s,\gamma ,\mu \right)=\left\{\begin{array}{cc} \sqrt{\frac{\gamma }{2\pi }}e^{-\frac{\gamma }{2(s-\mu )}}{\left(s-\mu \right)}^{-\frac{3}{2}}, & 0<\mu <s<\infty ,\\ 0, & s\leq 0, \end{array}\right.$$

where μ is the minimum step of the random walk and γ is a scale factor.

In this operator, to generate new individuals, a function $f_{\mathrm{shuffle}}:O\to O$ is used, which is defined as an index shuffler operator except for generating random numbers with a Lévy distribution. Specifically, it is necessary to evaluate the individuals who should receive genetic improvement before and after the procedure, and those who cannot show improvement should be replaced by new individuals generated with $f_{\mathrm{shuffle}}$. In detail, the steps that define the genetic improvement operator are presented in Algorithm 5 below.


**Algorithm 5** Population improvement with diversity maintenance.
**Input:**
c                  Representative individual*w*                 Genetic relevance vector$P_{\mathrm{Worst}}=\left\{x_{1},x_{2},\cdots ,x_{N_{\mathrm{Worst}}}\right\}$   The $N_{\mathrm{Worst}}$ worst individualsN×M              The JSSP dimension*F*                  Fitness function1: $P_{\mathrm{Improved}}:=\mathrm{Algorithm 4}\left(c,w,P_{\mathrm{Worst}},N,M\right)$ ▹ The set of individuals improved by the genetic improvement process is obtained through Algorithm 4.2: **for**
i=1 to $N_{\mathrm{Genes}}$**do**
▹ All improved individuals ${\hat{x}}_{i}$ should be evaluated to ensure that the fitness has improved.3:  $F_{\mathrm{no}}\;\mathrm{improvement}:=F\left(x_{i}\right)$            ▹$x_{i}$ is the original individual.4:  $F_{\mathrm{improvement}}:=F\left({\hat{x}}_{i}\right)$             ▹${\hat{x}}_{i}$ is the improved individual.5:  **if** $F_{\mathrm{no}}\;\mathrm{improvement}\leq F_{\mathrm{improvement}}$**then**    ▹ In case there is no improvement, the individual in question must be replaced by a new individual generated from the random permutation, with this being defined by the Lévy distribution, of a feasible solution.6:    $P_{\mathrm{Improved}}:=P_{\mathrm{Improved}}-\left\{{\hat{x}}_{i}\right\}.$  ▹ The individual from $P_{\mathrm{Improved}}$ is removed.7:    ${\hat{x}}_{i}:=f_{\mathrm{shuffle}}\left(\left(1,1,\cdots ,1,2,2,\cdots ,2,\cdots ,N,N,\cdots ,N\right)\right)$ ▹ The new individual is generated using the Lévy distribution.8:    $P_{\mathrm{Improved}}:=P_{\mathrm{Improved}}\cup \left\{{\hat{x}}_{i}\right\}.$ ▹ The generated individual assumes its position in $P_{\mathrm{Improved}}$.9:  **end if**10: **end if** **Output:**  $P_{\mathrm{Improved}}=\left\{{\hat{x}}_{1},{\hat{x}}_{2},\cdots ,{\hat{x}}_{N_{\mathrm{Worst}}}\right\}$  Improved individuals


### 4.4. Scheme of Use for Proposed Operators: Algorithm Structure

The proposed genetic improvement strategy was developed to be as versatile as possible in the sense that it can be attached to any GA-like method. Thus, the proposed operator must be used after the execution of the original operators of the method considered in order to guide solutions that were not able to stand out through the traditional strategies defined in the method. In other words, to use the proposed operator in a given GA-like method, we must obey the following steps:

1. Define the initial parameters and specifics of the chosen GA-like method.
2. Execute the operators that make up the GA-like method. These being, for example, the operators of crossover, mutation, local search, creation of new population, etc.
3. At the end of an iteration involving the traditional operators of the selected GA-like method, we make a sub-population $P_{\mathrm{Worst}}$ with the worst $N_{\mathrm{Worst}}$ individuals in the current population.
4. At the same time, we select the best $N_{\mathrm{Top}}$ individuals in the population to compose the representative individual.
5. Build the representative individual using the strategy described in **Stage 1** of Section 4.3.
6. Determine a relevance scale to the genes of the representative individual.
7. Conduct the genetic improvement of the $P_{\mathrm{Worst}}$ individuals using the most relevant $N_{\mathrm{Genes}}$ genes of the representative individual.
8. Replace in the current population of the method all individuals who obtained an improvement in the fitness value in the process of genetic improvement and return in the execution of the original operators of the considered GA-like method. Those who have not improved should be replaced by new individuals randomly generated according to Levy’s exponential distribution, following the procedure of Al-Obaidi and Hussein [55].

**Figure 5:**
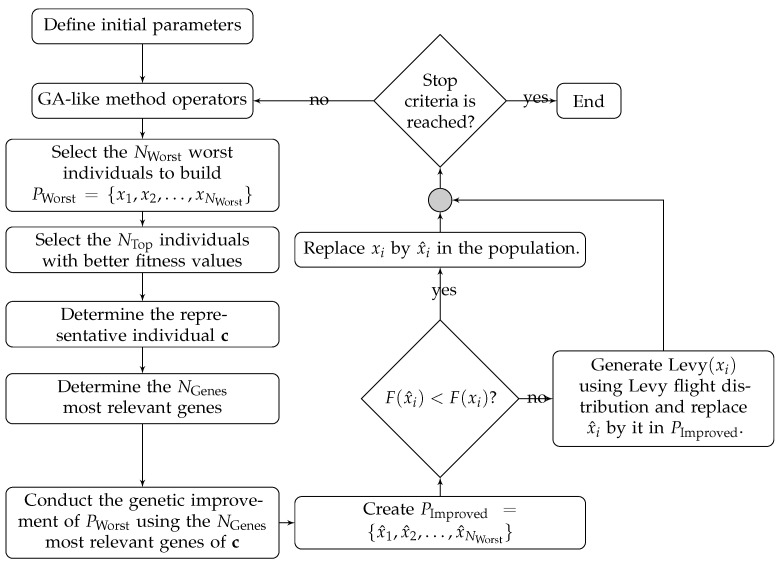
Flow chart of our proposed Genetic Improvement operator for Genetic Algorithm.

In Figure 5, we present a flowchart that illustrates the sequence of steps of the proposed genetic improvement process.

## 5. Implementation and Experimental Results

### 5.1. Experimental Environment

For the conduction of the experiments, we considered two different situations: in the first, we evaluated the impact that the proposed operator causes on five GA-like methods, all of which were obtained using the framework of Viana, Morandin Junior and Contreras [13], in eight JSSP instances of varying complexity; in the second, we compare with recent methods in the literature the ability of the proposed operator to look for good solutions in 58 instances of JSSP that compose the area benchmark, with 3 from Fisher and Thompson (FT) [45], 40 from Lawrence (LA) [46], 10 from Applegate and Cook (ORB) [56] and 5 from Adams, Balas and Zawack (ABZ) [57]. In detail, in this second situation, we consider relevant and recent methods which deal with the JSSP with the same specific instances and, when existing, presented in papers published in the last three years. In all, we consider for comparison the following methods: mXLSGA [13], NGPSO [44], SSS [43], GA-CPG-GT [32], DWPA [58], MeCSO [42], GWO [59], IPB-GA [31], NIMGA [60], aLSGA [20], PaGA [61]. The proposed algorithm is coded in MATLAB and we performed the evaluations on a computer with 2.4GHz Intel(R) Core i7 CPU and 16GB of RAM.

### 5.2. Results and Comparison with Other Algorithms

For the first testing situation, we consider five variations of the Viana, Morandin Junior and Contreras [13] framework: a basic GA (GA), GA with Search Area Adaptation (GSA) [30], GA with Local Search (LSGA) [29], GA with Elite Local Search and agent adjustment (aLSGA) [20] and GA with multi-crossover and massive local search (mXLSGA) [13]. In each of these versions that represent the state-of-the-art in GA-type techniques for JSSP solution, the proposed genetic improvement operator, GIFA, is added, and evaluations were performed on eight JSSP instances of varying complexity that compose the benchmark of the area, as 1 by Fisher and Thompson (1963) (FT) and 7 by Lawrence (1984) (LA): the FT 06, of dimension 6×6, and best known solution (BKS) equal to 55; the LA 01, of dimension 10×5, and the BKS is equal to 666; LA 06, of dimension 15×5, and the BKS is equal to 926; the LA 11, of dimension 20×5, and the BKS is equal to 1222; LA 16, of dimension 10×10, and the BKS is equal to 945; LA 23, of dimension 15×10, and the BKS is equal to 1032; LA 26, of dimension 20×10, and the BKS is equal to 1218; and LA 31, with a dimension of 30×10, and the BKS is equal to 1784. Thus, each GA-like method considered has a version with the proposed operator, represented by the acronym GIFA next to its standard acronym.

Our main purpose was to evaluate the impact of using GIFA in each of the GA-like methods; therefore, we kept the best possible configuration of each of the methods available in the original works, with the exception that everyone had 100 individuals in their populations, and we ran it for 100 generations. Furthermore, we added to each of them the configuration referring to GIFA, which is defined as follows: $N_{\mathrm{Top}}=N_{\mathrm{Worst}}=10$. It is worth noting that the choice for these last two parameters is not random. We experimentally verified that this would be the fairest possible common configuration when considering all the GA-like methods mentioned here. For that, we analyzed some specific performance metrics. In detail, let $S_{\Phi }\left(N_{\mathrm{Top}},N_{\mathrm{Worst}}\right)$ be the solution obtained by the method Φ without using the proposed operator and $S_{\Phi +GIFA}\left(N_{\mathrm{Top}},N_{\mathrm{Worst}}\right)$ the solution obtained using the GIFA operator, both of which use the same values for $N_{\mathrm{Top}}$ and for $N_{\mathrm{Worst}}$. Furthermore, we define ${\mathrm{Imp}}_{\Phi }\left(N_{\mathrm{Top}},N_{\mathrm{Worst}}\right)$ to be the improvement that using the GIFA operator gives to the Φ method considering $N_{\mathrm{Top}}$ and $N_{\mathrm{Worst}}$ as:

(4) $${\mathrm{Imp}}_{\Phi }\left(N_{\mathrm{Top}},N_{\mathrm{Worst}}\right):=\max\left\{\frac{S_{\Phi }\left(N_{\mathrm{Top}},N_{\mathrm{Worst}}\right)-S_{\Phi +GIFA}\left(N_{\mathrm{Top}},N_{\mathrm{Worst}}\right)}{S_{\Phi }\left(N_{\mathrm{Top}},N_{\mathrm{Worst}}\right)},0\right\}.$$

The objective is to analyze an average of improvement values ${\mathrm{Imp}}_{\Phi }\left(N_{\mathrm{Top}},N_{\mathrm{Worst}}\right)$ in several executions of the method on the same specific instance and on the same parameter configuration. Furthermore, we consider an average value for this improvement measure depending on the methods considered and the configuration given for the GIFA parameters: the value $\mathrm{AvgImp}(N_{\mathrm{Top}},N_{\mathrm{Worst}})$, defined in Equation (5):

(5) $$\mathrm{AvgImp}\left(N_{\mathrm{Top}},N_{\mathrm{Worst}}\right):=\frac{1}{5}\sum_{\Phi \in \{\mathrm{GA},\mathrm{GSA},\mathrm{LSGA},\mathrm{aLSGA},\mathrm{mXLSGA}\}}\frac{1}{N_{\mathrm{run}}}\sum_{i=1}^{N_{\mathrm{run}}}{\mathrm{Imp}}_{\Phi }^{i}\left(N_{\mathrm{Top}},N_{\mathrm{Worst}}\right),$$

where each method is executed $N_{\mathrm{run}}$ times and ${\mathrm{Imp}}_{\Phi }^{i}\left(N_{\mathrm{Top}},N_{\mathrm{Worst}}\right)$ represents the GIFA improvement with respect to *i*-th execution of that method.

**Figure 6:**
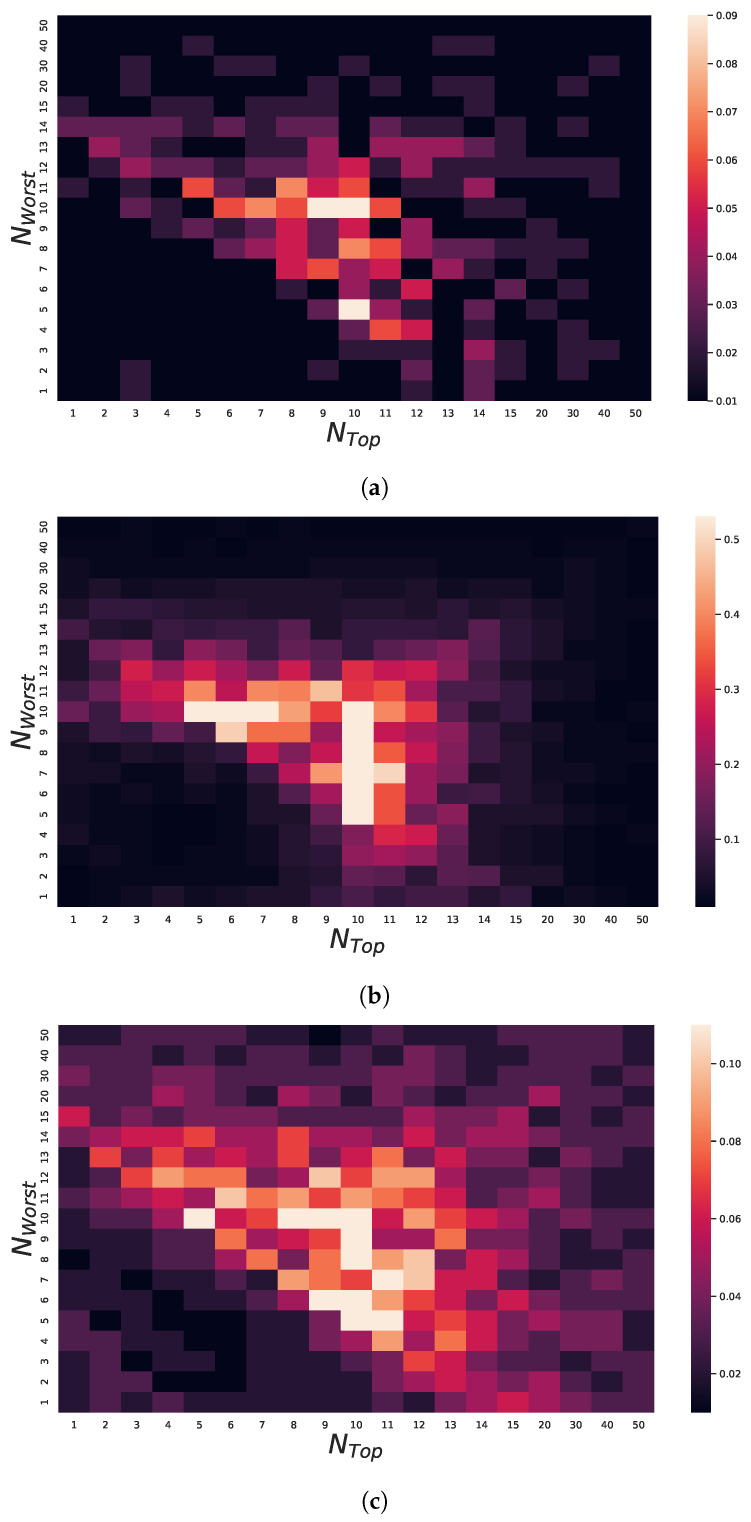
Experiments with respect to $N_{\mathrm{Top}}$ and $N_{\mathrm{Worst}}$ settings. In each heatmap, the average enhancement values $\mathrm{AvgImp}(N_{Top},N_{Worst})$ for $N_{\mathrm{Top}}$ and $N_{\mathrm{Worst}}$ are varying in grid on set {1,2,3,4,5,6,7,8,9,10,11,12,13,14,15,20,30,40,50}. For the computation of these values, $N_{\mathrm{run}}=35$ executions for each method were considered. (**a**) LA01. (**b**) LA21. (**c**) LA25.

In Figure 6, we represent through heatmaps the values of $\mathrm{AvgImp}(N_{\mathrm{Top}},N_{\mathrm{Worst}})$ calculated considering $N_{\mathrm{run}}=35$ runs of each GA -like method and with respect to three example bases: LA01, which consists of a simple instance; and instances LA21 and LA25, considered with high difficulty. In addition, the following set of possible $N_{\mathrm{Top}}$ and $N_{\mathrm{Worst}}$ configurations was considered for the creation of these images: {1,2,3,4,5,6,7,8,9,10,11,12,13,14,15,20,30,40,50}. In these conditions, we noticed that the use of the operator contributed with greater intensity in the more complex bases than in the simple base.

This is because the GA-Like methods can find good solutions for instance LA01 without the help of the operator; however, they have great difficulty finding good solutions for instances LA21 and LA25. Therefore, we noticed that the influence of the proposed operator is greater for more complex instances. Furthermore, we noticed a certain tendency in the sense that the operator is not able to positively influence the GA-like method when we choose small values, i.e., close to 1, and large values, greater than 15, for $N_{\mathrm{Top}}$ and $N_{\mathrm{Worst}}$. This is because very low values for these parameters reduce the functionality and influence of the operator since it has a low population sample, because $N_{\mathrm{Top}}$ is low and a low influence in poorly adapted individuals, as $N_{\mathrm{Worst}}$ is also low.

Furthermore, if the values assigned to these parameters are too large, the diversity of the population will be compromised, since a large portion of the population, defined by $N_{\mathrm{Worst}}$ individuals, will receive the genes defined by the other portion of the population, formed by $N_{\mathrm{Top}}$ individuals. For this reason we see a concentration of higher average $\mathrm{AvgImp}(N_{\mathrm{Top}},N_{\mathrm{Worst}})$ in the central regions of the heatmaps, that is when $N_{\mathrm{Top}}$ and $N_{\mathrm{Worst}}$ assume values close to 10 individuals. Therefore, we consider this configuration for the following analyses.

In this case, the best value, the worst value, the mean and the standard deviation (SD) of the makespan values calculated at 35 independent executions of each method on the eight JSSP instances considered are presented in Table 1. The number of times the method reached the best known solution is also presented (Number of optima); the number of iterations (Iteration of the Optimum) required to reach the best known solution; and the average time (Time (s)) in seconds that the technique takes to perform 100 iterations.

In Table 1, it is possible to observe that the operator made all methods more stable, reducing the amplitude of the mean and standard deviation in all situations in which improvement was possible. Furthermore, in most cases, the addition of the GIFA operator resulted in a decrease in the worst makespan value found. In fact, the operator was not able to improve this indicator only with respect to the mXLSGA method and considering three instances: LA 23, LA 26 and LA 31. An analogous phenomenon can be observed with respect to the best value obtained by each technique, since that, in most cases, the use of the GIFA operator makes the original technique able to reach a value closer to the best-known solution for the evaluated instance. In this case, it is possible to observe that the use of the GIFA operator increased the number of best-known solutions found by the techniques in all instances.

This fact is observed mainly in instances of lesser complexity. However, in more complex instances, specifically from the instance LA 16, the proposed operator was able to help a base technique to find the best-known solution only in the cases of the aLSGA and mXLSGA techniques, the latter being able to find these values without the use of the genetic improvement operator. This serves as an indication that the proposed operator offers a considerable increase in the stability of the method; however, the ability to explore the search space still has a strong dependence on the original technique. This occurs because GIFA guides individuals with makespan values considered bad in regions where individuals with good fitness values are known to exist, in order to increase local exploration and, therefore, find good solutions; however, it is up to the original technique to indicate good search regions. In addition, it is worth noting that the improvement that the GIFA operator provides to a base technique does not have much relation to the computational time required for its execution, since this is defined between 0.2 and 0.3 seconds, unlike the transgenic operator of [15], which requires an expensive preprocessing and simulation step to determine the genetic relevance.

Thus, the second situation considered should serve as an experiment in this sense so that we can evaluate the ability of the proposed operator to increase the search and exploration power of a given technique. For this, we added the proposed GIFA operator in a technique already known to be effective in finding good solutions in the JSSP instances that compose the benchmark today: the mXLSGA [13]. In this case, we evaluate GIFA-mXLSGA at 3 FT instances; 40 instances LA; 10 ORB instances; and 5 ABZ instances. In Table 2, Table 3, Table 4 and Table 5, we presented the results derived from 10 independent executions of our method on each instance. The columns indicate, respectively, the instance that was tested, the instance size (number of Jobs × number of Machines), the optimal solution of each instance, the results achieved by each method considering all the executions (best solution found and error percentage (Equation (6)) and the mean of the error with respect to each benchmark (MErr).

(6) $$E_{\%}=100\times \frac{\mathrm{Best}-\mathrm{BKS}}{\mathrm{BKS}},$$

in which $E_{\%}$ is the relative error, “BKS” is the best known Solution and “Best” is the best value obtained by executing the algorithm 10 times for each instance.

Analyzing Table 2, Table 3, Table 4 and Table 5, we can see that the proposed genetic improvement operator was able to improve the search capability of the mXLSGA method (the GA-like method with the best results for JSSP). Specifically, considering only the LA instances, the use of the proposed operator was able to reduce the magnitude of the mean relative error by 0.12, which corresponds to a reduction of 19.67% of its value. In other words, the GIFA operator made the mXLSGA method able to find the best known makespan in 72.5% of the LA instances, obtaining a mean relative error of 0.50, the lowest among all methods. With respect to FT instances, the proposed operator GIFA did not compromise the search capability of mXLSGA, causing the best known solutions to be found in all instances. In the case of ORB instances, GIFA improved the performance of mXLSGA in ORB05 instance and made the method capable of finding the best known solution in ORB06, reducing the average error of the technique from 0.54 to 0.46. Furthermore, with respect to the ABZ instances, the average error of GIFA-mXLSGA is less than half the error of mXLSGA, since the proposed genetic improvement operator improved the results of the base technique in the ABZ07 and ABZ09 instances. In summary, some points can be highlighted when analyzing the results referring to Table 2, Table 3, Table 4 and Table 5:

- There was no worsening of the results in any instance with the use of the GIFA operator;
- The GIFA-mXLSGA method had the smallest relative error;
- The GIFA operator made mXLSGA able to find the best known solution in instance LA 22;
- GIFA operator improved mXLSGA results in 7 LA instances;
- The GIFA operator made mXLSGA able to find the best known solution in instance ORB06 and improved the solution obtained in ORB05,
- The GIFA operator reduced the average error of mXLSGA by 53% in ABZ instances.

With these results, it can be seen that in the tested JSSP instances, the proposed genetic improvement operator is effective in increasing the efficiency of the mXLSGA base technique in finding good solutions.

## 6. Conclusions

To obtain advances in the solution of instances of the well-known JSSP, this work proposed the development of a genetic improvement operator based on the analysis of the frequency of genes present in well-adapted individuals of the population: the GIFA operator. This operator was proposed in a versatile way so that it can be easily integrated into any GA-like method. In this work, its performance was proven in 58 well-known JSSP instances of different complexities. The considered instances were FT [45], LA [46], ORB [56] and ABZ [57]. GIFA results were compared with other approaches in related works: mXLSGA [13], NGPSO [44], SSS [43], GA-CPG-GT [32], DWPA [58], GWO [59], IPB-GA [31], aLSGA [20], among others.

To evaluate GIFA’s performance, the operator was attached to five different GA-like metaheuristics that represent the state of the art in the specialized literature, with mXLSGA as one. All techniques and their versions with GIFA were performed 35 times, and some facts were observed. In this case, during the evaluations, we found that GIFA made all the metaheuristics more stable since it reduced the mean and standard deviation in all cases where this was possible.

In addition, the worst value presented by each technique during its executions was also reduced in most cases. Something similar occurred with the indicator of the best solution found with each technique. These facts corroborate the assumption that GIFA helps GA-like metaheuristics to find better solutions. However, the search capability of the GA-like method itself has a strong influence on the operator, as the operator guides poorly adapted individuals to good regions of the search space; however, it is up to the base technique to detect these regions. In the second situation, the ability of the proposed material to calculate good solutions for JSSP instances was evaluated and, for that, the obtained results with metaheuristics of the most varied types and inspirations were compared.

Thus, it was possible to observe that mXLSGA with GIFA presents a competitive search power compared to the works that comprise the state of the art on the subject. In this case, the method presented the smallest mean relative error in most situations considered, having surpassed all the techniques on which its components were based. This also serves as confirmation for the assumption that GIFA is capable of helping GA-like metaheuristics increase their search power.

Numerically, the GIFA operator was able to improve the relative error MErr of the mXLSGA method from 0.61% to 0.49% in the case of LA bases, from 0.54% to 0.46% in the case of ORB bases and from 4.43% to 2.07% in the case of ABZ bases, representing, respectively, improvements of 19.67%, 14.81% and 53.27% for that measure. Consequently, the average between the MErr of the mXLSGA considering all four test bases, corresponding to 1.395%, was reduced to 0.7555% with the use of the proposed operator—a reduction of 45.88% from the original value. Thus, we concluded that the proposed method is robust with the ability to obtain good results in instances of varied complexities, since GIFA-mXLSGA presented better or at least competitive results when compared with the other methods present in the specialized literature.

For future advances and developments, we intend to consider deep-learning techniques, mainly reinforcement-learning methods, to detect genetic influences on chromosomes from a GA-like method population. Furthermore, we intend to expand the developed material to other problems in the same field of application, such as Flexible Job Shop Scheduling [62] and to other classes of problems that demand combinatorial optimization, such as pseudo-colorization problems in graphs [21,22].

## Figures and Tables

**Table 1:** GA-like methods statistics for 35 executions of each method.

Instance	Method	Best	Worst	Mean	SD	Number of Optima	Iteration of the Optimum	Time (s)
FT06	GA	55	57	55.45	0.85	27	52	2.4
	GIFA-GA	55	56	55.14	0.35	30	38	2.63
	GSA	55	55	55	0	35	8	39.78
	GIFA-GSA	55	55	55	0	35	8	40.02
	LSGA	55	59	57.68	1.43	6	27	7.95
	GIFA-LSGA	55	56	55.83	0.38	6	20	8.17
	aLSGA	55	55	55	0	35	11	11.51
	GIFA-aLSGA	55	55	55	0	35	6	13.01
	mXLSGA	55	55	55	0	35	5	22.11
	GIFA-mXLSGA	55	55	55	0	35	5	22.37
LA01	GA	666	712	679.02	9.98	6	76	2.65
	GIFA-GA	666	678	669.37	5.17	15	26	2.94
	GSA	666	715	677.8	13.61	13	10	51.79
	GIFA-GSA	666	687	672.34	7.43	17	10	51.96
	LSGA	666	726	697	16.65	1	61	12.15
	GIFA-LSGA	666	707	688.67	15.59	8	23	12.42
	aLSGA	666	666	666	0	35	11	20.07
	GIFA-aLSGA	666	666	666	0	35	6	20.29
	mXLSGA	666	666	666	0	35	5	38.06
	GIFA-mXLSGA	666	666	666	0	35	4	38.34
LA06	GA	926	938	927.4	2.87	24	79	3.34
	GIFA-GA	926	936	926.86	2.26	30	54	3.57
	GSA	926	935	926.31	1.54	33	7	67.31
	GIFA-GSA	926	926	926	0	35	7	67.56
	LSGA	926	970	935.8	13.15	17	55	19.87
	GIFA-LSGA	926	952	932.28	9.08	20	41	20.13
	aLSGA	926	926	926	0	35	3	38.07
	GIFA-aLSGA	926	926	926	0	35	3	38.31
	mXLSGA	926	926	926	0	35	2	71.98
	GIFA-mXLSGA	926	926	926	0	35	2	72.21
LA11	GA	1222	1256	1235.97	10.81	5	58	3.97
	GIFA-GA	1222	1253	1233.48	9.52	6	51	4.18
	GSA	1222	1276	1232.14	14.94	20	19	81.59
	GIFA-GSA	1222	1263	1231.24	13.56	26	15	81.7
	LSGA	1222	1299	1251.6	19.62	2	32	31.33
	GIFA-LSGA	1222	1278	1250.17	11.09	4	26	31.59
	aLSGA	1222	1222	1222	0	35	5	60.82
	GIFA-aLSGA	1222	1222	1222	0	35	4	61.03
	mXLSGA	1222	1222	1222	0	35	3	116.57
	GIFA-mXLSGA	1222	1222	1222	0	35	3	116.84
LA16	GA	982	1100	1045.6	26.4	0	-	2.89
	GIFA-GA	982	1061	1022.89	20.51	0	-	3.12
	GSA	994	1110	1046.77	26.37	0	-	55.31
	GIFA-GSA	994	1021	1017.38	15.49	0	-	55.63
	LSGA	1016	1148	1084.25	32.27	0	-	20.62
	GIFA-LSGA	1016	1077	1037.11	26.62	0	-	20.83
	aLSGA	959	985	980.51	4.48	0	-	38.75
	GIFA-aLSGA	956	982	975.12	2.36	0	-	38.98
	mXLSGA	945	982	972.25	13.3	2	96	66.25
	GIFA-mXLSGA	945	979	959.93	6.37	7	49	66.51
LA23	GA	1189	1336	1271.71	34.44	0	-	3.78
	GIFA-GA	1151	1324	1269.51	30.95	0	-	4
	GSA	1148	1347	1214.08	43.85	0	-	73.19
	GIFA-GSA	1121	1339	1191.25	30.27	0	-	73.42
	LSGA	1214	1419	1295.34	43.7	0	-	38.39
	GIFA-LSGA	1115	1369	1278.36	34.79	0	-	38.63
	aLSGA	1035	1115	1078	16.34	0	-	75.62
	GIFA-aLSGA	1032	1098	1067.58	15.06	2	75	75.79
	mXLSGA	1032	1093	1060.45	17.96	1	51	123.64
	GIFA-mXLSGA	1032	1093	1039.8	16.54	2	34	123.87
LA26	GA	1525	1699	1619.51	39.5	0	-	4.44
	GIFA-GA	1485	1667	1614.11	32.59	0	-	4.65
	GSA	1433	1586	1512.22	36.47	0	-	91.67
	GIFA-GSA	1420	1523	1503.73	17.99	0	-	91.94
	LSGA	1517	1665	1597.94	36.67	0	-	60.84
	GIFA-LSGA	1492	1537	1534.11	28.74	0	-	61.07
	aLSGA	1302	1384	1343.28	19.69	0	-	124.93
	GIFA-aLSGA	1273	1382	1332.68	13.28	0	-	125.12
	mXLSGA	1218	1371	1300.85	41.88	6	85	203.95
	GIFA-mXLSGA	1218	1371	1258.51	32.32	11	67	204.3
LA31	GA	2120	2326	2223.14	48.45	0	-	6.23
	GIFA-GA	2111	2322	2197.31	45.19	0	-	6.51
	GSA	1943	2142	2050.17	63.09	0	-	130.65
	GIFA-GSA	1919	2101	1964.2	56.94	0	-	130.89
	LSGA	2005	2336	2177	66.29	0	-	123.23
	GIFA-LSGA	1986	2301	2119.88	62.49	0	-	123.57
	aLSGA	1808	1897	1843.51	21.17	0	-	258.53
	GIFA-aLSGA	1784	1861	1841.25	19.13	2	80	258.81
	mXLSGA	1784	1845	1807.71	19.2	5	80	424.56
	GIFA-mXLSGA	1784	1845	1805.98	18.96	7	71	424.77

**Table 2:** Comparison of computational results between GIFA-mXLSGA and other algorithms for FT. The symbol “-” means “not evaluated in that instance”.

Instance	Size	BKS	GIFA-mXLSGA		mXLSGA		NGPSO		SSS		GA-CPG-GT		GWO		IPB-GA		aLSGA	
			Best	$E_{\%}$	Best	$E_{\%}$	Best	$E_{\%}$	Best	$E_{\%}$	Best	$E_{\%}$	Best	$E_{\%}$	Best	$E_{\%}$	Best	$E_{\%}$
FT06	6×6	55	55	0.00	55	0.00	55	0.00	55	0.00	55	0.00	55	0.00	55	0.00	55	0.00
FT10	10×10	930	930	0.00	930	0.00	930	0.00	936	0.64	935	0.53	940	1.07	960	3.22	930	0.00
FT20	20×5	1165	1165	0.00	1165	0.00	1210	3.86	1165	0.00	1180	1.28	1178	1.11	1192	2.31	1165	0.00
MErr				**0.00**		**0.00**		**1.28**		**0.21**		**0.60**		**0.73**		**1.84**		**0.00**

**Table 3:** Comparison of computational results between GIFA-mXLSGA and other algorithms for LA. The symbol “-” means “not evaluated in that instance”.

Instance	Size	BKS	GIFA-mXLSGA		mXLSGA		NGPSO		SSS		GA-CPG-GT		DWPA		GWO		IPB-GA		aLSGA	
			Best	$E_{\%}$	Best	$E_{\%}$	Best	$E_{\%}$	Best	$E_{\%}$	Best	$E_{\%}$	Best	$E_{\%}$	Best	$E_{\%}$	Best	$E_{\%}$	Best	$E_{\%}$
LA01	10×5	666	666	0.00	666	0.00	666	0.00	666	0.00	666	0.00	666	0.00	666	0.00	666	0.00	666	0.00
LA02	10×5	655	655	0.00	655	0.00	655	0.00	655	0.00	655	0.00	655	0.00	655	0.00	655	0.00	655	0.00
LA03	10×5	597	597	0.00	597	0.00	597	0.00	597	0.00	597	0.00	614	2.84	597	0.00	599	0.33	606	1.50
LA04	10×5	590	590	0.00	590	0.00	590	0.00	590	0.00	590	0.00	598	1.35	590	0.00	590	0.00	593	0.50
LA05	10×5	593	593	0.00	593	0.00	593	0.00	593	0.00	593	0.00	593	0.00	593	0.00	593	0.00	593	0.00
LA06	15×5	926	926	0.00	926	0.00	926	0.00	926	0.00	926	0.00	926	0.00	926	0.00	926	0.00	926	0.00
LA07	15×5	890	890	0.00	890	0.00	890	0.00	890	0.00	890	0.00	890	0.00	890	0.00	890	0.00	890	0.00
LA08	15×5	863	863	0.00	863	0.00	863	0.00	863	0.00	863	0.00	863	0.00	863	0.00	863	0.00	863	0.00
LA09	15×5	951	951	0.00	951	0.00	951	0.00	951	0.00	951	0.00	951	0.00	951	0.00	951	0.00	951	0.00
LA10	15×5	958	958	0.00	958	0.00	958	0.00	958	0.00	958	0.00	958	0.00	958	0.00	958	0.00	958	0.00
LA11	20×5	1222	1222	0.00	1222	0.00	1222	0.00	1222	0.00	1222	0.00	1222	0.00	1222	0.00	1222	0.00	1222	0.00
LA12	20×5	1039	1039	0.00	1039	0.00	1039	0.00	-	-	1039	0.00	1039	0.00	1039	0.00	1039	0.00	1039	0.00
LA13	20×5	1150	1150	0.00	1150	0.00	1150	0.00	-	-	1150	0.00	1150	0.00	1150	0.00	1150	0.00	1150	0.00
LA14	20×5	1292	1292	0.00	1292	0.00	1292	0.00	-	-	1292	0.00	1292	0.00	1292	0.00	1292	0.00	1292	0.00
LA15	20×5	1207	1207	0.00	1207	0.00	1207	0.00	-	-	1207	0.00	1273	5.46	1207	0.00	1207	0.00	1207	0.00
LA16	10×10	945	945	0.00	945	0.00	945	0.00	947	0.21	946	0.10	993	5.07	956	1.16	946	0.10	946	0.10
LA17	10×10	784	784	0.00	784	0.00	794	1.27	-	-	784	0.00	793	1.14	790	0.76	784	0.00	784	0.00
LA18	10×10	848	848	0.00	848	0.00	848	0.00	-	-	848	0.00	861	1.53	859	1.29	853	0.58	848	0.00
LA19	10×10	842	842	0.00	842	0.00	842	0.00	-	-	842	0.00	888	5.46	845	0.35	866	2.85	852	1.18
LA20	10×10	902	902	0.00	902	0.00	908	0.66	-	-	907	0.55	934	3.54	937	3.88	913	1.21	907	0.55
LA21	15×10	1046	1052	0.57	1059	1.24	1183	13.09	1076	2.86	1090	4.20	1105	5.64	1090	4.20	1081	3.34	1068	2.10
LA22	15×10	927	927	0.00	935	0.86	927	0.00	-	-	954	2.91	989	6.68	970	4.63	970	4.63	956	3.12
LA23	15×10	1032	1032	0.00	1032	0.00	1032	0.00	-	-	1032	0.00	1051	1.84	1032	0.00	1032	0.00	1032	0.00
LA24	15×10	935	940	0.53	946	1.17	968	3.52	-	-	974	4.17	988	5.66	982	5.02	1002	7.16	966	3.31
LA25	15×10	977	984	0.71	986	0.92	977	0.00	-	-	999	2.25	1039	6.34	1008	3.17	1023	4.70	1002	2.55
LA26	20×10	1218	1218	0.00	1218	0.00	1218	0.00	-	-	1237	1.55	1303	6.97	1239	1.72	1273	4.51	1223	0.41
LA27	20×10	1235	1261	2.10	1269	2.75	1394	12.87	1256	1.70	1313	6.31	1346	8.98	1290	4.45	1317	6.63	1281	3.72
LA28	20×10	1216	1239	1.89	1239	1.89	1216	0.00	-	-	1280	5.26	1291	6.16	1263	3.86	1288	5.92	1245	2.38
LA29	20×10	1152	1190	3.29	1201	4.25	1280	11.11	-	-	1247	8.24	1275	10.67	1244	7.98	1233	7.03	1230	6.77
LA30	20×10	1355	1355	0.00	1355	0.00	1355	0.00	-	-	1367	0.88	1389	2.50	1355	0.00	1377	1.62	1355	0.00
LA31	30×10	1784	1784	0.00	1784	0.00	1784	0.00	1784	0.00	1784	0.00	1784	0.00	1784	0.00	1784	0.00	1784	0.00
LA32	30×10	1850	1850	0.00	1850	0.00	1850	0.00	-	-	1850	0.00	1850	0.00	1850	0.00	1851	0.05	1850	0.00
LA33	30×10	1719	1719	0.00	1719	0.00	1719	0.00	-	-	1719	0.00	1719	0.00	1719	0.00	1719	0.00	1719	0.00
LA34	30×10	1721	1721	0.00	1721	0.00	1721	0.00	-	-	1725	0.23	1788	3.89	1721	0.00	1749	1.62	1721	0.00
LA35	30×10	1888	1888	0.00	1888	0.00	1888	0.00	-	-	1888	0.00	1947	3.125	1888	0.00	1888	0.00	1888	0.00
LA36	15×15	1268	1295	2.12	1295	2.12	1408	11.04	1304	2.83	1308	3.15	1388	9.46	1311	3.39	1334	5.20	-	-
LA37	15×15	1397	1407	0.71	1415	1.28	1515	8.44	-	-	1489	6.58	1486	6.37	-	-	1467	5.01	-	-
LA38	15×15	1196	1246	4.18	1246	4.18	1196	0.00	-	-	1275	6.60	1339	11.95	-	-	1278	6.85	-	-
LA39	15×15	1233	1258	2.02	1258	2.02	1662	34.79	-	-	1290	4.62	1334	8.19	-	-	1296	5.10	-	-
LA40	15×15	1222	1243	1.71	1243	1.71	1222	0.00	1252	2.45	1252	2.45	1347	10.22	-	-	1284	5.07	-	-
MErr				**0.49**		**0.61**		**2.42**		**0.59**		**1.50**		**3.52**		**1.27**		**1.99**		**0.80**

**Table 4:** Comparison of computational results between GIFA-mXLSGA and other algorithms for ORB. The symbol “-” means “not evaluated in that instance”.

Instance	Size	BKS	GIFA-mXLSGA		mXLSGA		IPB-GA		NIMGA		aLSGA		PaGA		LSGA	
			Best	$E_{\%}$	Best	$E_{\%}$	Best	$E_{\%}$	Best	$E_{\%}$	Best	$E_{\%}$	Best	$E_{\%}$	Best	$E_{\%}$
ORB01	10 × 10	1059	1068	0.85	1068	0.85	1099	3.78	1059	0	1092	3.12	1149	8.5	1088	2.74
ORB02	10 × 10	888	889	0.11	889	0.11	906	2.03	890	0.23	894	0.68	929	4.62	921	3.72
ORB03	10 × 10	1005	1023	1.79	1023	1.79	1056	5.07	1026	2.09	1029	2.39	1129	12.34	1041	3.58
ORB04	10 × 10	1005	1005	0	1005	0	1032	2.69	1019	1.39	1016	1.09	1062	5.67	1052	4.68
ORB05	10 × 10	887	887	0	889	0.23	909	2.48	893	0.68	901	1.58	936	5.52	903	1.8
ORB06	10 × 10	1010	1013	0.3	1019	0.89	1038	2.77	1012	0.2	1028	1.78	1060	4.95	1062	5.15
ORB07	10 × 10	397	397	0	397	0	411	3.53	397	0	405	2.02	416	4.79	408	2.77
ORB08	10 × 10	899	907	0.89	907	0.89	917	2	909	1.11	914	1.67	1010	12.35	908	1
ORB09	10 × 10	934	940	0.64	940	0.64	-		942	0.86	943	0.96	994	6.42	980	4.93
ORB10	10 × 10	944	944	0	944	0	-		-				-		-	
MErr				**0.46**		**0.54**		**3.04**		**0.73**		**1.7**		**7.24**		**3.37**

**Table 5:** Comparison of computational results between GIFA-mXLSGA and other algorithms for ABZ. The symbol “-” means “not evaluated in that instance”.

Instance	Size	BKS	GIFA-mXLSGA		mXLSGA		GA-CPG-GT		MeCSO		IPB-GA	
			Best	$E_{\%}$	Best	$E_{\%}$	Best	$E_{\%}$	Best	$E_{\%}$	Best	$E_{\%}$
ABZ05	10 × 10	1234	1234	0	1234	0	1238	0.32	1236	0.16	1241	0.57
ABZ06	10 × 10	943	943	0	943	0	947	0.42	949	0.64	964	2.23
ABZ07	20 × 15	656	657	0.15	695	5.95	-	-	-	-	719	9.6
ABZ08	20 × 15	648	713	10.03	713	10.03	-	-	-	-	738	13.89
ABZ09	20 × 15	679	680	0.15	721	6.19	-	-	-	-	742	9.28
MErr				**2.07**		**4.43**		**0.37**		**0.4**		**7.11**

## References

[1] Pardalos P.M., Du D.Z., Graham R.L. (2013). Handbook of Combinatorial Optimization.

[2] Sbihi A., Eglese R.W. (2010). Combinatorial optimization and green logistics. Ann. Oper. Res..

[3] James J., Yu W., Gu J. (2019). Online vehicle routing with neural combinatorial optimization and deep reinforcement learning. IEEE Trans. Intell. Transp. Syst..

[4] Matyukhin V., Shabunin A., Kuznetsov N., Takmazian A. Rail transport control by combinatorial optimization approach. Proceedings of the 2017 IEEE 11th International Conference on Application of Information and Communication Technologies (AICT).

[5] Ehrgott M., Gandibleux X. (2003). Multiobjective combinatorial optimization—Theory, methodology, and applications. Multiple Criteria Optimization: State of the Art Annotated Bibliographic Surveys.

[6] Parente M., Figueira G., Amorim P., Marques A. (2020). Production scheduling in the context of Industry 4.0: Review and trends. Int. J. Prod. Res..

[7] Groover M.P. (2007). Fundamentals of Modern Manufacturing: Materials Processes, and Systems.

[8] Hart E., Ross P., Corne D. (2005). Evolutionary scheduling: A review. Genet. Program. Evolvable Mach..

[9] Xhafa F., Abraham A. (2008). Metaheuristics for Scheduling in Industrial and Manufacturing Applications.

[10] Wu Z., Sun S., Yu S. (2020). Optimizing makespan and stability risks in job shop scheduling. Comput. Oper. Res..

[11] Wang L., Cai J.C., Li M. (2016). An adaptive multi-population genetic algorithm for job-shop scheduling problem. Adv. Manuf..

[12] Mhasawade S., Bewoor L. A survey of hybrid metaheuristics to minimize makespan of job shop scheduling problem. Proceedings of the 2017 International Conference on Energy, Communication, Data Analytics and Soft Computing (ICECDS).

[13] Viana M.S., Morandin Junior O., Contreras R.C. (2020). A Modified Genetic Algorithm with Local Search Strategies and Multi-Crossover Operator for Job Shop Scheduling Problem. Sensors.

[14] Viana M.S., Morandin Junior O., Contreras R.C. (2020). An Improved Local Search Genetic Algorithm with Multi-Crossover for Job Shop Scheduling Problem. Proceedings of the International Conference on Artificial Intelligence and Soft Computing.

[15] Viana M.S., Morandin Junior O., Contreras R.C. (2020). Transgenic Genetic Algorithm to Minimize the Makespan in the Job Shop Scheduling Problem. Proceedings of the 12th International Conference on Agents and Artificial Intelligence—Volume 2: ICAART. INSTICC.

[16] Lu Y., Huang Z., Cao L. (2020). Hybrid immune genetic algorithm with neighborhood search operator for the Job Shop Scheduling Problem. IOP Conf. Ser. Earth Environ. Sci..

[17] Milovsevic M., Lukic D., Durdev M., Vukman J., Antic A. (2016). Genetic algorithms in integrated process planning and scheduling—A state of the art review. Proc. Manuf. Syst..

[18] Çaliş B., Bulkan S. (2015). A research survey: Review of AI solution strategies of job shop scheduling problem. J. Intell. Manuf..

[19] Demirkol E., Mehta S., Uzsoy R. (1998). Benchmarks for shop scheduling problems. Eur. J. Oper. Res..

[20] Asadzadeh L. (2015). A local search genetic algorithm for the job shop scheduling problem with intelligent agents. Comput. Ind. Eng..

[21] Contreras R.C., Morandin Junior O., Viana M.S. (2020). A New Local Search Adaptive Genetic Algorithm for the Pseudo-Coloring Problem. Advances in Swarm Intelligence.

[22] Viana M.S., Morandin Junior O., Contreras R.C. (2020). An Improved Local Search Genetic Algorithm with a New Mapped Adaptive Operator Applied to Pseudo-Coloring Problem. Symmetry.

[23] Zang W., Ren L., Zhang W., Liu X. (2018). A cloud model based DNA genetic algorithm for numerical optimization problems. Future Gener. Comput. Syst..

[24] Li X., Gao L. (2016). An effective hybrid genetic algorithm and tabu search for flexible job shop scheduling problem. Int. J. Prod. Econ..

[25] Sastry K., Goldberg D., Kendall G. (2005). Genetic algorithms. Search Methodologies.

[26] do Amaral L.R., Hruschka E.R. Transgenic, an operator for evolutionary algorithms. Proceedings of the 2011 IEEE Congress of Evolutionary Computation (CEC).

[27] do Amaral L.R., Hruschka E.R. (2014). Transgenic: An evolutionary algorithm operator. Neurocomputing.

[28] Viana M.S., Contreras R.C., Junior O.M. (2021). A New Genetic Improvement Operator Based on Frequency Analysis for Genetic Algorithms Applied to Job Shop Scheduling Problem. Proceedings of the International Conference on Artificial Intelligence and Soft Computing.

[29] Ombuki B.M., Ventresca M. (2004). Local search genetic algorithms for the job shop scheduling problem. Appl. Intell..

[30] Watanabe M., Ida K., Gen M. (2005). A genetic algorithm with modified crossover operator and search area adaptation for the job-shop scheduling problem. Comput. Ind. Eng..

[31] Jorapur V.S., Puranik V.S., Deshpande A.S., Sharma M. (2016). A promising initial population based genetic algorithm for job shop scheduling problem. J. Softw. Eng. Appl..

[32] Kurdi M. (2019). An effective genetic algorithm with a critical-path-guided Giffler and Thompson crossover operator for job shop scheduling problem. Int. J. Intell. Syst. Appl. Eng..

[33] Liang X., Du Z. Genetic Algorithm with Simulated Annealing for Resolving Job Shop Scheduling Problem. Proceedings of the 2020 IEEE 8th International Conference on Computer Science and Network Technology (ICCSNT).

[34] Anil Kumar K.R., Das E.R., Yang L.J., Haq A.N., Nagarajan L. (2020). Genetic Algorithm and Particle Swarm Optimization in Minimizing MakeSpan Time in Job Shop Scheduling. Proceedings of ICDMC 2019.

[35] Zhang J., Cong J. Research on job shop scheduling based on ACM-GA algorithm. Proceedings of the 2021 IEEE 5th Advanced Information Technology, Electronic and Automation Control Conference (IAEAC).

[36] Wang B., Wang X., Lan F., Pan Q. (2018). A hybrid local-search algorithm for robust job-shop scheduling under scenarios. Appl. Soft Comput..

[37] Frausto-Solis J., Hernández-Ramírez L., Castilla-Valdez G., González-Barbosa J.J., Sánchez-Hernández J.P. (2021). Chaotic Multi-Objective Simulated Annealing and Threshold Accepting for Job Shop Scheduling Problem. Math. Comput. Appl..

[38] Zhou G., Zhou Y., Zhao R. (2021). Hybrid social spider optimization algorithm with differential mutation operator for the job-shop scheduling problem. J. Ind. Manag. Optim..

[39] Liu C. (2021). An improved Harris hawks optimizer for job-shop scheduling problem. J. Supercomput..

[40] Jiang T. (2018). A Hybrid Grey Wolf Optimization for Job Shop Scheduling Problem. Int. J. Comput. Intell. Appl..

[41] Dao T.K., Pan T.S., Pan J.S. (2018). Parallel bat algorithm for optimizing makespan in job shop scheduling problems. J. Intell. Manuf..

[42] Semlali S.C.B., Riffi M.E., Chebihi F. (2019). Memetic chicken swarm algorithm for job shop scheduling problem. Int. J. Electr. Comput. Eng..

[43] Hamzadayı A., Baykasoğlu A., Akpınar Ş. (2020). Solving combinatorial optimization problems with single seekers society algorithm. Knowl.-Based Syst..

[44] Yu H., Gao Y., Wang L., Meng J. (2020). A Hybrid Particle Swarm Optimization Algorithm Enhanced with Nonlinear Inertial Weight and Gaussian Mutation for Job Shop Scheduling Problems. Mathematics.

[45] Fisher C., Thompson G. (1963). Probabilistic learning combinations of local job-shop scheduling rules. Industrial Scheduling.

[46] Lawrence S. (1984). Resouce Constrained Project Scheduling: An Experimental Investigation of Heuristic Scheduling Techniques (Supplement).

[47] Croes G.A. (1958). A method for solving traveling-salesman problems. Oper. Res..

[48] Meng X., Liu Y., Gao X., Zhang H. (2014). A new bio-inspired algorithm: Chicken swarm optimization. Proceedings of the International Conference in Swarm Intelligence.

[49] Alkhateeb F., Abed-alguni B.H., Al-rousan M.H. (2022). Discrete hybrid cuckoo search and simulated annealing algorithm for solving the job shop scheduling problem. J. Supercomput..

[50] Xiong H., Shi S., Ren D., Hu J. (2022). A survey of job shop scheduling problem: The types and models. Comput. Oper. Res..

[51] Bierwirth C., Mattfeld D.C., Kopfer H. (1996). On permutation representations for scheduling problems. Proceedings of the International Conference on Parallel Problem Solving from Nature.

[52] Wegner P. (1960). A Technique for Counting Ones in a Binary Computer. Commun. ACM.

[53] Yang X.S., Deb S. (2013). Multiobjective cuckoo search for design optimization. Comput. Oper. Res..

[54] Yang X.S. (2010). Engineering Optimization: An Introduction with Metaheuristic Applications.

[55] Al-Obaidi A.T.S., Hussein S.A. (2016). Two improved cuckoo search algorithms for solving the flexible job-shop scheduling problem. Int. J. Perceptive Cogn. Comput..

[56] Applegate D., Cook W. (1991). A computational study of the job-shop scheduling problem. ORSA J. Comput..

[57] Adams J., Balas E., Zawack D. (1988). The shifting bottleneck procedure for job shop scheduling. Manag. Sci..

[58] Wang F., Tian Y., Wang X. A Discrete Wolf Pack Algorithm for Job Shop Scheduling Problem. Proceedings of the 2019 5th International Conference on Control, Automation and Robotics (ICCAR).

[59] Jiang T., Zhang C. (2018). Application of grey wolf optimization for solving combinatorial problems: Job shop and flexible job shop scheduling cases. IEEE Access.

[60] Kurdi M. (2016). An effective new island model genetic algorithm for job shop scheduling problem. Comput. Oper. Res..

[61] Asadzadeh L., Zamanifar K. (2010). An agent-based parallel approach for the job shop scheduling problem with genetic algorithms. Math. Comput. Model..

[62] Xie J., Gao L., Peng K., Li X., Li H. (2019). Review on flexible job shop scheduling. IET Collab. Intell. Manuf..

